# Characterization of mesenchymal stem cells from human dental pulp, preapical follicle and periodontal ligament

**Published:** 2013-03

**Authors:** Ali Reza Navabazam, Fatemeh Sadeghian Nodoshan, Mohammad Hasan Sheikhha, Sayyed Mohsen Miresmaeili, Mehrdad Soleimani, Farzaneh Fesahat

**Affiliations:** 1 *Department of Oral and Maxillo-facial Surgery, Dental School, Shahid Sadoughi University of Medical Sciences, Yazd, Iran.*; 2 *Research and Clinical Center for Infertility, Shahid Sadoughi University of Medical Sciences, Yazd, Iran.*; 3 *Yazd Academic Center of Education, Culture and Research Higher Education Institute, Yazd, Iran.*

**Keywords:** *Dental pulp*, *Preapical follicle*, *Mesenchymal stem cell*, *Periodontal ligament stem cells*

## Abstract

**Background: **Human dental stem cells have high proliferative potential for self-renewal that is important to the regenerative capacity of the tissue.

**Objective** **: **The aim was to isolate human dental pulp stem cells (DPSC), periodontal ligament stem cells (PDLSC) and periapical follicle stem cells (PAFSC) for their potential role in tissue regeneration.

**Methods & Materials: **In this experimental study, the postnatal stem cells were isolated from dental pulp, preapical follicle and periodontal ligament .The cells were stained for different stem cell markers by immunocytochemistry. To investigate the mesenchymal nature of cells, differentiation potential along osteoblastic and adipogenic lineages and gene expression profile were performed. For proliferation potential assay, Brdu staining and growth curve tests were performed. Finally, all three cell types were compared together regarding their proliferation, differentiation and displaying phenotype.

**Results:**The isolated cell populations have similar fibroblastic like morphology and expressed all examined cell surface molecule markers. These cells were capable of differentiating into osteocyte with different capability and adipocyte with the same rate. PAFSCs showed more significant proliferation rate than others. Reverse transcriptase PCR (RT-PCR) for nanog, oct4, Alkaline phosphatase (ALP) and glyceraldehydes-3-phosphate dehydrogenease (GADPH) as control gene showed strong positive expression of these genes in all three isolated cell types.

**Conclusion:** PDLSCs, DPSCs and PAFSCs exist in various tissues of the teeth and can use as a source of mesenchymal stem cells for developing bioengineered organs and also in craniomaxillofacial reconstruction with varying efficiency in differentiation and proliferation.

## Introduction

The human tooth is consisted of enamel, dentin, pulp and periodontium, all of these parts are developed by epithelial mesenchymal interactions, whereas mesenchymal cells within the dental papilla are responsible for formation of tooth pulp (1-3). PDL rise from the dental follicle (4). Current treatments regarding dental restorations based on autologous tissue grafts or metallic implants have some disadvantages such as; insufficient biocompatibility, resorption of bone, limited graft quantity, and donor-site morbidity. Knowledge of stem cells has created promising technique for regenerative dentistry, solving these problems and will replace damaged teeth or metal implants in future (5-8). Recently, several studies have been focused on isolation and characterization of human mesenchymal stem cell (MSCs) populations from dental tissues of extracted teeth and have evaluated differentiation abilities into many types of lineages, both in vivo and in vitro conditions or on types of scaffolds for dental tissue regeneration (9-19).

Many studies showed that there are postnatal stem cells in various tissues, such as bone marrow, skin and dental tissues in human as well as experimental animal models which can be used in therapeutic applications (5-8). Also, these cells demonstrate attractive valences for self-renewal and extension into different tissues and many of them have reported the usefulness of stem cell regenerative therapy tissue engineering which maybe good candidates for replacing missing teeth (19-21). 

It seems these sources of MSCs have some advantages over others even embryonic stem cells (ESCs). We can use tissues from teeth extracted for orthodontic aims or from third molar extracted teeth easily with safe accessibility without additional injury, and ethical problems. There are acceptable results in preliminary clinical trials with no problems with rejection by the host immune system (19, 22). 

Additionally, comparison of the differences or similarities between the various stem cell types was one of the basic approaches to understand the generation mechanism of these differences and their impact on biological and clinical processes. The main goal of this study was to isolate human dental DPSC, PDLSC and PAFSC from the developing root of impacted third molar and investigation of some aspects of differentiation, proliferation as well as gene expression in these cell lines.

## Materials and methods

**Isolation of stem cells and cell culture**

The normal and nondecayed human third molars were extracted for orthodontic treatment purposes from 20 adults (15-32 years of age) under sufficient informed consent at the Department of Oral and Maxillo-facial Surgery, Dental School of Shahid Sadoughi University of Medical Sciences, Yazd, Iran and it was approved by the ethics committee at the Yazd Research and Clinical Center for Infertility. 

PDL cells were scraped from third molars and the pulp tissue was gently separated from the crown and root. Tooth germs at the root-forming stage were obtained and named periapical follicles. These were removed from the root dentin with a scalpel (23-24). The tissue segments were digested in a solution of 3mg/mL collagenase type I and 4mg/ml dispase dissolved in DMEM for 1 hour at CO_2_ incubator. Then, single-cell suspensions were separated by passing the cells through a 70-mm cell strainer (Falcon, BD Labware, Franklin Lakes, NJ). The suspensions of cells were cultured into 25 cm^2^ plastic flask with alpha modification of Eagle’s medium (alpha- MEM, Gibco BRL, Carlsbad, CA) supplemented with 10% fetal bovine serum (FBS, Gibco BRL), 100U/mL penicillin, 100 mg/mL streptomycin (Gibco BRL) and amphotericin B (BIOCHROM AG) for primary culture and then incubated at 37^o^C .

**Proliferation potential assay**

To detect proliferation abilities, two experimental assays were performed in vitro. The proliferation rate of cells (P3) was determined with Brdu staining kit (Invitrogen, Carlsbad, CA, 92008 USA). The seeded cells were cultured in four slide chambers for 3 days to get conference. Then, cells were washed with PBS and fixed in paraformaldehide 4% and stained according to kit procedure for cultured cells (25). 

The numbers of positive proliferative cells were counted (500 nuclei) using a Brdu assay kit. For proliferation potential assay, PDLSC, DPSC and PAFSC at the 3^rd^ passage were cultured separately in 6-well plate for 1, 2, 3 and 4 days with optimal culture medium. Population growth curve of stem cells was determined every day after culturing up to 4 days. 

**In vitro differentiation**

Three-passage cells were plated at100 cell/Cm^2^ in a 25 Cm^2 ^flask, then this was incubated in DMEM supplemented with 100 IU/ml penicillin (Sigma Chemical Co.), 100 mg/ml streptomycin (Sigma Chemical Co), and 15% FCS for 4 days until confluence was achieved. For adipogenic differentiation, cells were plated into 6-well plates (P3; 2, 500 cells/Cm^2^) and cultured with proliferation normal media. After 70% confluency of the cells, the media was replaced with differentiation medium consisting of alpha-MEM, supplemented with 50 mg/ml indomethacine (Sigma Chemical Co.) and 100nM dexamethasone (Sigma Chemical Co.) for 3 weeks. 

The medium was changed every 2-4 days. Intracellular lipid vacuoles were detected by staining with 0.5% Oil Red O (Sigma- Aldrich) in methanol. For osteogenic differentiation, cells (P3; 2, 500 cells/Cm^2^) were plated in 6-well plates. The differentiation medium [alpha-MEM supplemented with 50 mg/ml ascorbic acid2-phosphate (Sigma Chemical Co.), 10nM dexamethasone (Sigma Chemical Co.), 10mM b-glycerophosphate (Sigma Chemical Co.) and 10% fetal bovine serum] was changed with the culture media with medium changes three times per week. After 3 weeks, osteogenic differentiation was examined by Alizarin red staining (Sigma Chemical Co) to do this, the cells were fixed with 10% formalin for 10 min and then stained with Alizarin red (2%, pH 4.2) for 45 seconds (1, 5).

**Immunocytochemistry**

Isolated putative stem cells from the PDL, dental pulp and periapical follicle (PDLSC, DPSC and PAFSC) were seeded on four-chamber slides (2*10^4^ cells/well; NUNC, Denmark) and cultured for 24 hours. After being washed in phosphate-buffered saline (PBS, pH 7.4) and fixed in 4% p-formaldehyde for 15 minutes, the samples were incubated with STRO-1 antibody (1:200; Invitrogen, Zymed laboratories), c-kit (1:200; Santa Cruz Biotechnology INC), Nanog (1:200; Santa Cruz Biotechnology INC),CD44 antibody (1:200; abcam), CD34 antibody (1:200; abcam) and CD31 antibody (1:200; abcam) for 3 hours. The cells were subsequently incubated with goat secondary antibodies of antimouse IgG-Cy3 (Zymed Laboratories, Carlsbad, CA), goat secondary antibodies of antimouse IgG-FITC (Sigma Aldrich, Int.), Rabbit IgG Rhodamine (Chemicon, Temecula, CA) and also goat secondary antibodies of antimouse IgG-FITC (Sigma Aldrich, Int.) for CD markers for 1 hour. Then the nuclei were stained in DAPI (2 mg/mL) for 10 minutes.

**Reverse transcriptase polymerase chain reaction (RT-PCR)**

cDNA was synthesized using a cells to cDNA^TM^ II kit (Ambion, the RNA Company, USA). The primer set for PCR included Alkaline phosphatase (ALP);F: CCT AAA AGG GCA GAA GAA GGA C, R: TCC ACC TAG GAT CAC ATC AAT G with an amplicon of 444 bp, OCT4; F: GAA GCT GGA GAA GGA GAA GCT, R: CAA GGG CCG CAG CTT ACA CAT with an amplicon of 243 bp, NANOG; F: CTC CTT CCA TGG ATC TGC TTA TTC, R: AGG TCT TCA CCT GTT TGT AGC TGA G with an amplicon of 265 bp and glyceraldehydes-3-phosphatedehydrogenease (GAPDH); F: AGC CGC ATC TTC TTT TGC GTC, R: TCA TAT TTG GCA GGT TTT TCT with an amplicon of 815 bp for internal control. The PCR reactions were pre-incubated in a PCR Master cycler gradient (Eppendorf, Hamburg, Germany) at 95^o^C for 5 minutes and then cycled 33 times at 95^o^C/30 sec, 55^o^C/45 sec, and 72^o^C/60 sec, followed by a final 10-minute extension at 72^o^C. The products were separated by electrophoresis on a 1.5% agarose gel and visualized by ultraviolet induced fluorescence.

**Statistical analysis**

All experiments were repeated three times. Results are expressed as themean standard deviation. Data were statistically analyzed using the one-way analysis of variance (ANOVA) and Tukey test was applied for post-Hoc comparison. Statistical significance was set at p<0.05.

## Results

**Isolation and** **immunocytochemistry** **of dental stem cells**

**Figure 1:**
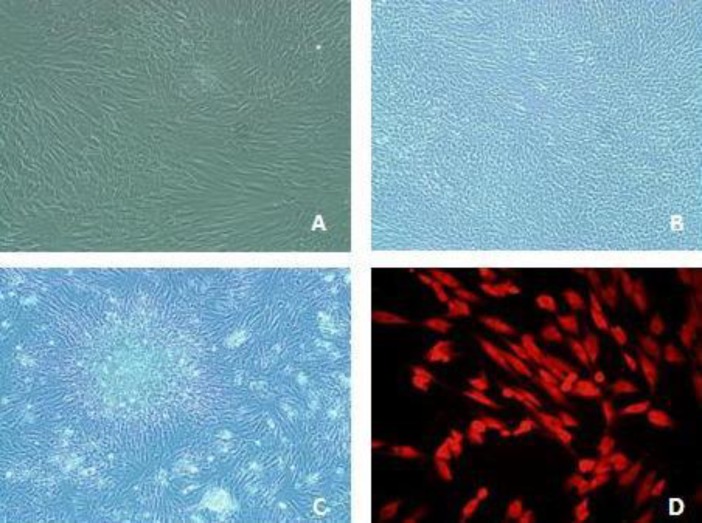
Postnatal dental stem cells were isolated from human dental tissues and identified as stem cells by immunocytochemistry.

The isolated cells had fibroblastic spindle shape morphology in cultured flasks (Figure 1A-C).The isolated cell populations expressed the cell surface molecule STRO-1 as an MSCs marker (Figure 1E). They also expressed other MSCs markers such asCD34, CD31, CD44, c-kit and nanog (Figure 5).

**Proliferation assays**

**Figure 2.**
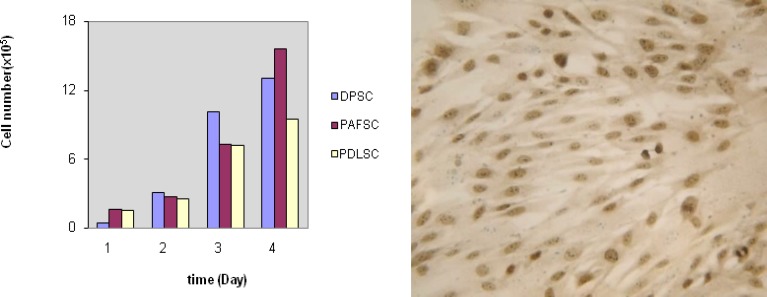
Dental stem cells were highly proliferative and produced comparable colonies in their culture condition.

In growth curve, PAFSC showed a higher proliferation rate at 4 days and a higher number of population growth compared to the others. DPSCs also were close to the PAFSC in proliferation rate (Figure 2a). Also, quantitative results of Brdu assay showed that the number of proliferative cells in the PAFSC (83±2.2%) rats was significantly higher than that in PDLSC (71±1.5%) and DPSC (67.40±1.2%) group (p=0.027) (Figure 2b).

**Differentiation potential of dental stem cells**

Dental stem cells that were grown in osteo inductive medium for 21 days demonstrated the capacity to form red mineralizing areas with a high level of calcium. There were sparsely scattered deposits from DPSC and PAFSC over the adherent layer, whereas extensive sheets of calcified deposits throughout the adherent layer were observed in PDLSC cultures after 2 weeks of induction (Figure 4A-C). In addition, staining observations of Alizarin red after 21 days of induction showed that the accumulations of calcium levels in cultures of PDLSC were higher than the other cultured cells. In other hand, the accumulated calcium level in DPSC was the same as PDLSC. To investigate the ability of dental stem cells to differentiate into adipocyte, the cells were cultured with adipo inductive medium for three weeks. Oil red O positive droplets were detected in all of seeding cells (Figure 4D-E). This result showed that this stem cell could differentiate into other phenotypes although developmentally are varied.

**Expression of pluripotent stem cells markers**

**Figure 3:**
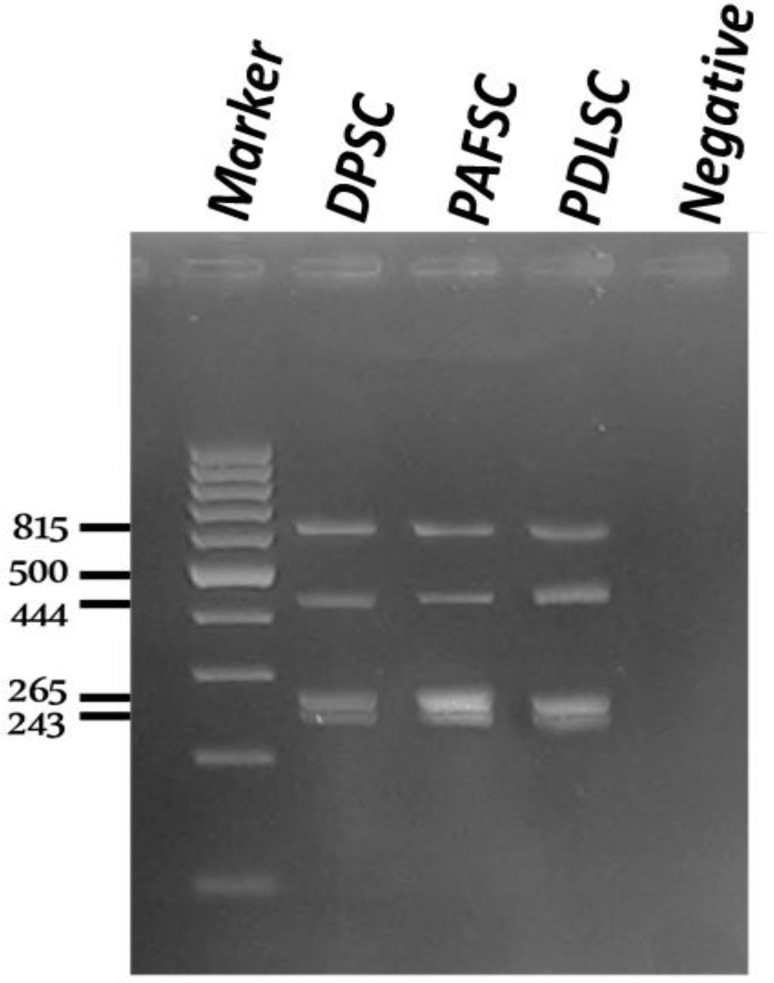
Expression of stem cell markers

PDLSC, DPSC and PAFSC were analysed by RT-PCR for undifferentiated, pluripotent stem cell markers including Oct-4 and Nanog. All dental stem cells showed high levels of expression of these markers (Figure 3). GAPDH and water were used as an internal and negative control for all samples, respectively. In addition, the gene expression of alkaline phosphatase was positive as odontoblast marker at undifferentiated dental stem cells.

Stem cells were isolated from different tissues had a fibroblastic spindle shape at PDLSC(A), DPSC(B) and PAFSC(C) at passage 3.. They also exressed STRO-1(D), an MSC marker.

A) Population growth curve of dental pulp stem cells (DPSC), periodontal ligament stem cells (PDLSC) and periapical follicle stem cells (PAFSC). The total number of isolated dental stem cells was counted every day after seeding with 1×10^4^ cells.

B) Brdu assay of PAFSC showed high numbers of proliferative stem cells (brown nucleus) comparing other cell types. Note the specific nuclear labelling by DAB .10× magnification.

**Figure 4:**
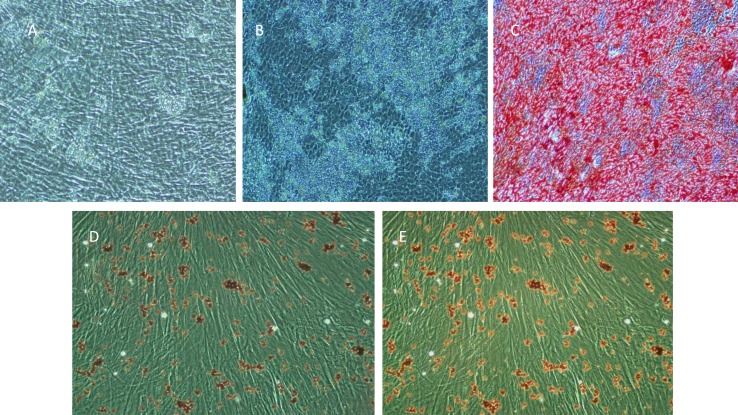
Differentiation of dental stem cells. PAFSCs (A) in 1 and 3 weeks (B) after induction by osteogenic condition media

**Figure 5.**
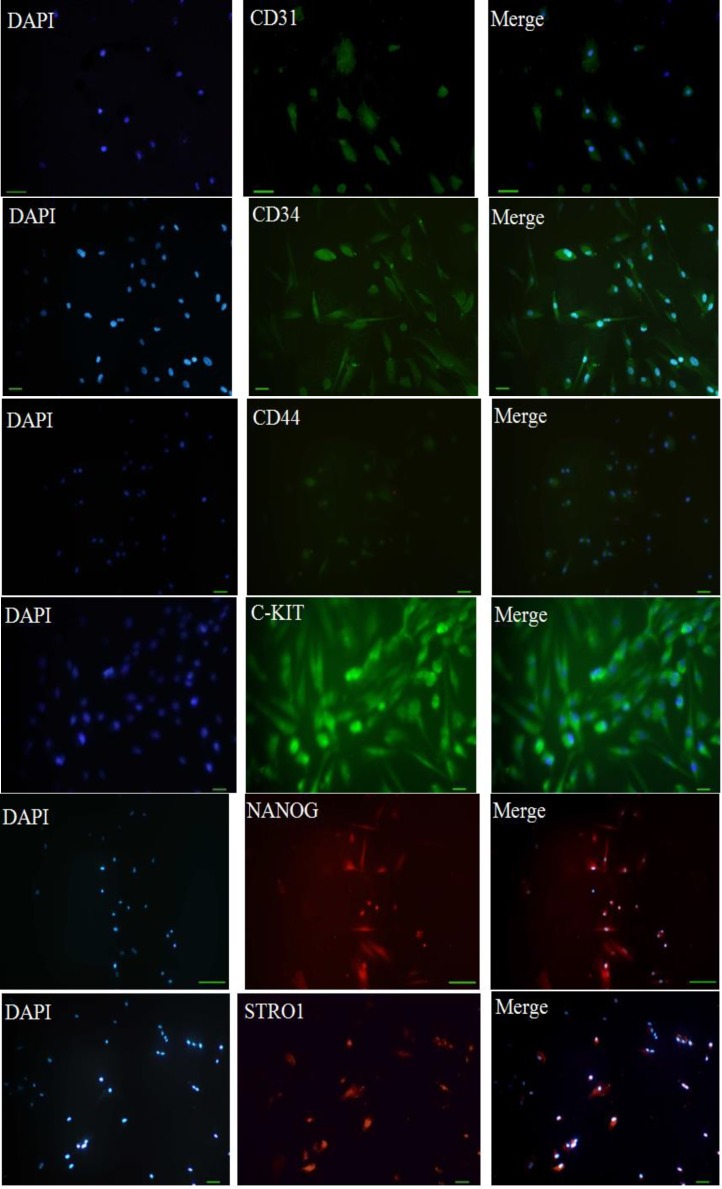
Phenotypic and molecular characterization of dental stem cells

## Discussion

In this study, we successfully isolated and identified 3 types of stem cell populations (DPSCs, PDLSCs, and PAFSCs) derived from each dental tissue of impacted or extracted nondecayed human third molars. Recently, many studies have isolated MSCs with self-renewal and multipotent capabilities from different teeth obtained during dental treatment and characterized clogenicity and differentiation capacity into multiple cell lineages in vitro and in vivo conditions (23, 25-28, 30, 31). We compared these stem cell populations with each other for differential and proliferation potentials and gene expression profiles. PAFSCs have much higher proliferative abilities than DPSCs, PDLSCs, based on evaluations of Brdu staining assay and population growth curves. 

Yang *et al* also reported the same results about proliferation rate and differentiation capacity at early passage that this is probably due to a difference between sources of obtaining cell types where PAFSC are undifferentiated cells derived from the developing tissue (32). PDLSC makes many calcium nodules in the osteogenic culture medium despite of capability to differentiate into adipocytes which was suggested by Gronthos *et al* and Zhang *et al* (23, 31). 

This finding is associated with an upregulation in the expression of ALP, as detected by RT-PCR. DPSCs and PAFSCs are also capable of differentiating into osteoblasts/ odontoblasts and adipocytes. In those studies, they have evaluated and compared PDLSCs and PAFSCs characteristics in different passages in vitro whereas we did not perform this work in our study (23, 31, 32). They showed morphology of these cell populations would be changed and their stem cell properties can be lost during different passages. 

Some researchers believe that dental stem cells (DSCs) present in extracted human teeth have pluripotent properties like ESCs or induced pluripotent stem cells (iPSCs) that are valuable tools in routine dental therapeutics. They are considered to possess very little possibility of developing into tumors, unlike ESCs and iPSCs (33-35). For example, immature dental pulp stem cells, which are derived from dental pulp of exfoliated deciduous teeth, has been introduced to express several pluripotent cell markers (36). 

Therefore DSCs can use as an alternative stem cell population even to make a biocompatible tooth (20, 21). furthermore, mesenchymal nature of DPSC, PDLSC and PAFSC such as their expression of STRO-1,a specific markers for MSCs, and other CD markers may implied the beneficial approaches of regenerative dental therapy, stem cell therapy and tooth replacement therapy according to Rodríguez-Lozano *et al* and others (11, 19, 22).

To conclude, PDLSCs, DPSCs and PAFSCs are present in various tissues of the teeth and can use as a source of MSCs for developing bioengineered organs and also in craniomaxillofacial renovation with varying efficiency in differentiation and proliferation. Knowing the different aspects of DSCs is important for using them to produce an effective bioengineered tooth or for the management of dental diseases in patients. Future studies are probably needed focusing on subpopulations of stem cells derived from teeth characterized by specific surface markers, their distinctive properties and might be autologous stem cell transplantation for using them in clinical applications.

## References

[1] Chung IH, Yamaza T, Zhao H, Choung PH, Shi S, Chai Y (2009). Stem cell property of post migratory cranial neural crest cells and their utility in alveolar bone regeneration and tooth development. Stem Cells.

[2] Rothová M, Feng J, Sharpe PT, Peterková R, Tucker AS (2011). Contribution of mesoderm to the developing dental papilla. Int J Dev Biol.

[3] Pispa J, Thesleff I (2003). Mechanisms of ectodermal organogenesis. Dev Biol.

[4] Nakashima M, Reddi AH (2003). The application of bone morphogeneticproteins to dental tissue engineering. Nature.

[5] YY Jo, HJ Lee, SY Kook, HW Choung, JY Park, JH Chung (2007). Isolation and Characterization of Postnatal Stem Cellsfrom Human Dental Tissues. Tissue Eng.

[6] Khalili MA, Anvari M, Hekmati-Moghadam SH, Sadeghian-Nodoushan F, Fesahat F, Miresmaeili SM (2011). Therapeutic Benefit of Intravenous Transplantation of Mesenchymal Stem Cells after Experimental Subarachnoid Hemorrhage in Rats. J Stroke Cerebrovasc Dis.

[7] JH Chung, PH Choung, KT Lim, HV Choung (2012). Scaffolds for Human Dental Stem Cells to Regenerate Cementum. Stem Cells and Cancer Stem Cells.

[8] Blau HM, Brazelton TR, Weimann JM (2001). The evolving concept of a stem cell: entity or function?. Cell.

[9] Huang GT, Gronthos S, Shi S (2009). Mesenchymal stem cells derivedfrom dental tissues vs those from other sources: their biology and role in regenerative medicine. J Dent Res.

[10] Shi S, Bartold PM, Miura M, Seo BM, Robey PG, Gronthos S (2005). The efficacy of mesenchymal stem cells to regenerate and repair dental structures. Orthod Craniofac Res.

[11] Tirino V, Paino F, Rosa A, Papaccio G (2012). Identification, Isolation, Characterization, and Banking of Human Dental Pulp Stem Cells. Methods in Molecular Biology.

[12] Yalvac ME, Ramazanoglu M, Tekguc M, Bayrak OF, ShaWgullina AK, Salafutdinov II (2010). Human tooth germ stem cells preserve neuroprotective effects after long-term cryo-preservation. Curr Neurovasc Res.

[13] Karaoz E, Okcu A, Gacar G, Saglam O, Yuruker S, Kenar H (2010). AComprehensive characterization study of human bone marrowMSCs with an emphasis on molecular and ultrastructural properties. J Cell Physiol.

[14] Gandia C, Arminan A, Garcia-Verdugo JM, Lledo E, Ruiz A, Minana MD (2008). Human dental pulpstem cells improve left ventricular function, induce angiogenesis,and reduce infarct size in rats with acute myocardial infarction. Stem Cells.

[15] Gronthos S, Brahim J, Li W, Fisher LW, Cherman N, Boyde A (2002). Stem cell properties of human dental pulp stem cells. J Dent Res.

[16] d’Aquino R, Graziano A, Sampaolesi M, Laino G, Pirozzi G, De RosaA (2007). Human postnatal dental pulp cells co-differentiateinto osteoblasts and endotheliocytes: a pivotal synergyleading to adult bone tissue formation. Cell Death Differ.

[17] Laino G, d’ Aquino R, Graziano A, Lanza V, Carinci F, Naro F (2005). A new population of human adult dental pulp stem cells: a useful source of living autologous fibrous bone tissue (LAB). J Bone Miner Res.

[18] Huang AH, Chen YK, Lin LM, Shieh TY, Chan AW (2008). Isolationand characterization of dental pulp stem cells from a super numerary tooth. J Oral Pathol Med.

[19] Rodríguez-Lozano FJ, Insausti CL, Iniesta F, Blanquer M, Ramírez MC, Meseguer L (2012). Mesenchymal dental stem cells in regenerative dentistry. Med Oral Patol Oral Cir Bucal.

[20] Yamada Y (2004). Autogenous injectable bone for regeneration with mesenchymal stem cells and platelet-rich plasma: tissueengineeredbone regeneration. Tissue Eng.

[21] Deng W, Han Q, Liao L, Li C, Ge W, Zhao Z (2005). Engrafted bone marrowderivedFlk-1‏mesenchymal stem cells regenerate skin tissue. Tissue Eng.

[22] Fawzy El-Sayed KM, Dörfer C, Fändrich F, Gieseler F, Moustafa MH, Ungefroren H (2012). Adult Mesenchymal Stem Cells Explored in the Dental Field. Adv Biochem Eng Biotechnol.

[23] Seo BM, Miura M, Gronthos S, Bartold PM, Batouli S, Brahim J (2004). Investigation of multipotent postnatal stem cells from humanperiodontal ligament. Lancet.

[24] Gronthos S, Mankani M, Brahim J, Robey PG, Shi S (2000). Postnatal human dental pulp stem cells (DPSCs) in vitro and in vivo. Proc Natl Acad Sci.

[25] Ellwart E, Dormer P (1985). Effect of 5-Fluro-2-deoxyuridine (FdUrd) on 5-bromo-2deoxyuridine (BrdUrd) incorporation into DNA measured with monoclonal BrdUrd antibody and by the BrdUrd/Hoechst quenching effect. Cytometry.

[26] Miura M, Gronthos S, Zhao M, Lu B, Fisher LW, Robey PG (2003). SHED: stem cells from human exfoliated deciduous teeth. Proc Natl Acad Sci.

[27] Sonoyama W, Liu Y, Fang D, Yamaza T, Seo BM, Zhang C (2006). Mesenchymal stem cell-mediated functional tooth regeneration in swine. PLo SOne.

[28] Morsczeck C, Gotz W, Schierholz J, Zeilhofer F, Kuhn U, Mohl C (2005). Isolation of precursor cells (PCs) from human dental follicle of wisdom teeth. Matrix Biol.

[29] Ding G, Liu Y, Wang W, Wei F, Liu D, Fan Z (2010). Allogeneic periodontal ligament stem cell therapy for periodontitis in swine. Stem Cells.

[30] Iohara K, Zheng L, Ito M, Ishizaka R, Nakamura H, Into T (2009). Regeneration of dental pulp after pulpotomy by transplantation of CD31(-)/CD146(-) side population cells from a canine tooth. Regen Med.

[31] Zhanga J, Ana Y, Gaoa LN, Zhangb YJ, Jinb Y, Chen FM (2012). The effect of aging on the pluripotential capacity and regenerative potential of humanperiodontalligamentstemcells. Biomaterials.

[32] Han C, Yang Z, Zhou W, Jin F, Song Y, Wang Y (2010). Periapical Follicle Stem Cell: A Promising Candidate for Cementum/Periodontal Ligament Regeneration and Bio-Root Engineering. Stem Cells and Development.

[33] Hilfiker A, Kasper C, Hass R, Haverich A (2011). Mesenchymal stem cells and progenitor cells in connective tissue engineering and regenerative medicine: is there a future for transplantation?. Langenbecks Arch Surg.

[34] Nakahara T, Nakamura T, Kobayashi E, Kuremoto K, MatsunoT, Tabata Y (2004). In situ tissue engineering of periodontal tissues by seeding with periodontal ligament-derived cells. Tissue Eng.

[35] Kuroda Y, Kitada M, Wakao S, Nishikawa K, Tanimura Y, Makinoshima H (2010). Unique multipotent cells in adult human mesenchymal cell populations. Proc Natl Acad Sci USA.

[36] Zhao M, Amiel SA, Ajami S, Jiang J, Rela M, Heaton N (2008). Amelioration of streptozotocin-induced diabetes in mice with cells derived from human marrow stromal cells. PLo SOne.

